# Account of a Case in Which a Stone, Formed in One of the Kidneys, Was Extracted through an Abscess in the Back

**Published:** 1797

**Authors:** Herman Schützercrants


XXIV. *Account of a Case in which a Stone, formed in one of the Kidneys, was extracted through an Abscess in the Back. By* Herman Schützercrants, *M. D.* Vide *Kongl. Vetenskaps nya Handlingar*, Tom. XII. 8vo. Stockholm, 1791.

THE patient, whose case is here described, was a widow, aged fifty-six years, who, for several months, had been subject to violent pains in her back and loins, when our author was first called to her assistance, in consultation with her physician, Dr. Lindberg, who had prescribed for her different remedies, but without out being able to relieve her complaints. This was in Auguſt, 1777. She had then a quick full pulſe, with alternate ſhiverings and heat, and other ſymptoms of fever; and was ſubject to frequent vomiting. She complained alſo of a ſwelling which was found to extend over the whole of the right hypochondrium. This ſwelling was ſoft and œdematous, without any diſcoloration of the integuments.

Venæſection, purgatives, opiates, and other remedies adapted to the ſymptoms, were had recourſe to, but without procuring any permanent relief to the patient. At the end of three weeks, from the time our author firſt ſaw her, the ſwelling was conſiderably increaſed, and the pain and feveriſh ſymptoms were become more violent. Inſtead of being every where ſoft, as at firſt, there was a conſiderable hardneſs to be felt in the tumour, immediately under the ſhort ribs, and when this part was preſſed with the finger, the patient complained of great pain.

Different plaſters and cataplaſms were applied to this tumour, with a view to promote ſuppuration: and in the courſe of ten or twelve days more, a fluctuation could be diſtinctly felt. The integuments now became gradually thinner and and more pointed, and the abfcefs being at length opened with a biftoury, more than a quart of matter was difcharged from it.

Our author introduced a probe into the abfcefs, but was unable to afcertain the full extent of it. Suitable dreffings and bandages were applied to the part, and for fome days there was a good difcharge from the abfcefs, and the pain and feverifh fymptoms fubfided; but at the end of a fortnight the difcharge became thin and even watery, wetting the compreffes through, excoriating the integuments, and diffufing a fmell of urine. Our author was now convinced that this difcharge came from the kidney; but ftill, he obferves, he had no fufpicion of a ftone, till, on introducing a probe again into the abfcefs, he felt it in contact with a hard, rough, and feemingly broad fubftance. The orifice of the abfcefs being now too fmall to admit his finger, he enlarged it with a biftoury fufficiently for that purpofe; but ftill he was only juft able to reach the ftone with the end of his finger. By means of a pair of forceps, however, he at length fucceeded in extracting the calculus delineated in Plate II. fig. vi.

After the extraction of this ftone, the difcharge of urine from the abfcefs gradually diminifhed; miniſhed; and at the end of a fortnight entirely ceaſed. The wound now began to yield a laudable pus; the patient's ſtrength was ſupported by Peruvian bark, and the wound gradually healing, ſhe at length found herſelf free from complaint.

From inquiry of the patient, our author learnt, that previouſly to this complaint ſhe had been ſubject to the Gout; but had never obſerved any ſtone or gravel in her urine.

This caſe, he obſerves, proves, that the extraction of a ſtone from the kidneys, or what is called nephrotomy, is, as different writers have already remarked, practicable; but it can, he thinks, be adviſable only in inſtances like the preſent, when matter formed within the kidney is able to make its way to the ſurface of the body, and thus points out to the ſurgeon the tract he has to purſue.

Dr. Schützercrants refers his readers for ſome uſeful obſervations on this ſubject*, to a

* The reader will alſo find an inſtance, by the writer of this note, of urinary calculi making their way from the kidney through a fiſtulous abſceſs in the loins, inſerted in the Philoſophical Tranſactions, Vol. LXIV. Part I. for the year 1775. Editor.

paper by Mr. Hevin, entitled *Recherches historiques et critiques sur la Nephrotomie, ou Taille du Rein,* inserted in *Memoires de l'Academie de Chirurgie,* Tom. III. p. 238. 4to. Paris, 1751.

